# Initial experience with HydroPearl microspheres for uterine artery embolization for the treatment of symptomatic uterine fibroids

**DOI:** 10.1186/s42155-021-00223-9

**Published:** 2021-03-31

**Authors:** Matthew A. Patetta, Ari J. Isaacson, Jessica K. Stewart

**Affiliations:** Division of Vascular and Interventional Radiology, Department of Radiology, University of North Carolina School of Medicine, 2017 Old Clinic Bldg CB #7510, Chapel Hill, NC 27599-7510 USA

**Keywords:** UAE, Hydropearl, Microspheres, Embolization, Uterine fibroids

## Abstract

**Background:**

Uterine Artery Embolization (UAE) is a minimally invasive procedure used to treat symptomatic uterine fibroids. The HydroPearl Microsphere (Terumo Interventional Systems) is an embolic agent approved for UAE and other embolization procedures. The purpose of this article is to describe our initial experience with HydroPearl for UAE in patients with symptomatic uterine fibroids. Twenty-one patients who underwent UAE using HydroPearl Microspheres at a single institution from May 1, 2018 to December 31, 2019 were included in the study. The electronic medical record (EMR) was reviewed for documentation of short- and long-term complications, as well as improvements in menorrhagia and bulk-type symptoms. We also describe unique attributes of the HydroPearl Microsphere that should be considered when utilizing this embolization particle for UAE.

**Results:**

Of the 21 patients, 18 had a 3-month or later post-procedure follow-up documented in the EMR and were included in the analysis. The average time between the UAE procedure and the most recent clinical note was 145 days. Sixteen patients reported symptoms of menorrhagia and 13 reported bulk symptoms prior to the UAE procedure. On follow-up, 13/16 patients (81%) and 12/13 patients (92%) experienced improvement in menorrhagia and bulk symptoms, respectively. The only recorded complication was amenorrhea in 4 patients (22%) who had an average age of 51 years.

**Conclusions:**

Several characteristics of HydroPearl Microsphere may prove helpful when considering these embolic particles for use in UAE. Our initial experience with this embolic agent suggest that the reatment response for menorrhagia and bulk symptoms are largely similar to success rates reported in the literature for other embolic agents. Larger studies are needed to evaluate the safety and efficacy of this embolic particle for this indication.

## Background

Uterine artery embolization (UAE) is a minimally-invasive, image-guided procedure that is widely accepted as a safe and effective treatment for symptomatic uterine leiomyomata, also known as uterine fibroids (Spencer et al., 2013). Several meta-analyses have demonstrated superior patient outcomes for UAE compared to surgical management, including faster recovery time, shorter hospital stay, fewer major complications, and higher patient satisfaction rates (Jun et al., 2012; van der Kooij et al., 2011; Sandberg et al., 2018). UAE involves the administration of embolic agents (such as gelfoam, non-spherical polyvinyl alcohol, or spherical embolization particles) into the bilateral uterine arteries to deprive the uterine fibroids of blood supply, resulting in necrosis and shrinkage of the uterine fibroids (Spencer et al., 2013; Dariushnia et al., 2014).

To further refine this procedure, novel embolic agents with different properties that could be superior to current embolics should be investigated. The HydroPearl Microsphere (Terumo Interventional Systems, Tokyo, Japan) is a tightly calibrated spherical embolic particle made of polyethylene glycol that is approved in the United States for UAE and other embolization procedures. While some early positive clinical data are available regarding the safety and efficacy of HydroPearl Microspheres for prostate artery embolization and embolization of the shoulder, no data are available for this embolic agent for UAE (Bagla et al., 2020). The purpose of this article is to describe our initial experience with this embolic agent for UAE for symptomatic uterine fibroids.

## Methods

### Subjects

This retrospective study was reviewed and approved by the Institutional Review Board (IRB). The IRB approved a waiver of informed consent and HIPAA Authorization under 45 CFR 46.116(d) and 45 CFR 164.512(i) (Jun et al., 2012)(ii), respectively. This study included 21 patients who underwent UAE using HydroPearl Microsphere embolization particles for the treatment of symptomatic uterine fibroids at a single academic institution from May 1, 2018 to December 31, 2019. One interventional radiologist with 3 years of experience performed all procedures.

### Uterine artery embolization procedure

UAE was completed as described in the literature (Spencer et al., 2013; Dariushnia et al., 2014). Patients received 3 g Ampicillin/Sulbactam IV, 4 mg ondansetron IV, and 1 g of acetaminophen IV prior to the procedure. Arterial access was via the left radial artery under ultrasound guidance. A 5-Fr vertebral catheter was advanced over a guidewire and positioned just above the aortic bifurcation, and an aortogram was performed in order locate the iliac arteries. A superior hypogastric nerve block was then performed for post-procedure pain control, as described in the literature, at the level of either L4 or L5 depending on the patient’s arterial anatomy (Yoon et al., 2018). The 5-Fr catheter was then advanced into the uterine artery, and digital subtraction angiography was performed. In some cases, a microcatheter was inserted and advanced to beyond the horizontal segment of the uterine artery to minimize the risk of non-target embolization of cervicovaginal arterial branches. The HydroPearl embolization particles were then prepared by adding saline and contrast to the vial of particles and gently agitating the vial until the particles were well suspended. For 600-μm HydroPearl particles, approximately 3 mL of contrast (Omnipaque 300, GE Healthcare, Chicago, IL) and 1.5 mL of saline were added to the vial of particles. For 800-μm HydroPearl particles, approximately 3 mL of contrast and 2.5 mL of saline were added to the vial. For 1100-μm HydroPearl particles, approximately 3 mL of contrast and 3 mL of saline were added to the particles. The embolic was allowed to rest for 2 min prior to embolization to optimize suspension of the particles, and the amounts of contrast and saline added to the vial were adjusted to optimize suspension if needed. The embolic was then administered into the uterine artery under fluoroscopic observation until the solution lingered for 3–5 cardiac cycles within the uterine artery. Embolization was initially performed using 600-μm +/− 75-μm HydroPearl particles, with subsequent upsizing to 800-μm and 1100-μm HydroPearl particles as needed to achieve the desired endpoint. After embolization was completed, the process was repeated for the contralateral uterine artery. All but two patients were discharged the day of the procedure. Patients were discharged with ketorolac PO for 3 days, ibuprofen for the following 3 days, oxycodone as needed for breakthrough pain, ondansetron as needed, docusate sodium, and levofloxacin for 5 days. Acetaminophen was used in lieu of ibuprofen and ketorolac for one patient who had renal insufficiency.

### Follow-up and data collection and analysis

After the procedure, patients had clinical follow-up with the interventional radiologist either by telephone or in clinic. Standard follow-up protocol included a phone call to the patient approximately 1 week post-procedure and clinic follow-up appointments 1 and 3 months post-procedure. Additional follow-up clinic visits or telephone encounters were completed on an as-needed basis.

The clinical documents in the EMR were reviewed for evidence of adverse events following UAE. These were categorized using the Society of Interventional Radiology (SIR) adverse event classification (Khalilzadeh et al., 2017). The clinic notes were also reviewed to determine whether the patients undergoing UAE were being treated for menorrhagia, bulk-type symptoms, or both. Bulk symptoms were defined as pelvic pain, fullness, heaviness, bloating, severe cramping, dyspareunia, urinary frequency or urgency, and constipation. The most recent clinical note in the EMR was reviewed to determine whether there was documentation of improvement in bleeding and/or bulk symptoms.

## Results

Of the 21 patients, three patients were excluded because they did not have clinical follow-up at least 3 months post-procedure either by telephone or in clinic. Characteristics and procedural data for the remaining 18 patients are presented in Table 1. The average patient age was 45.3 years. All 18 patients had technically successfully UAE procedures, defined as at least one uterine artery successfully embolized. The embolization was performed via radial arterial access in 17/18 patients (94.4%). One patient was converted from radial to femoral arterial access due to radial artery spasm (Patient 13). The bilateral uterine arteries were embolized in all patients except two: patient 3 had a dominant left uterine artery supplying a single large fibroid, and a diminutive right uterine artery; patient 12 had an enlarged right uterine artery; the left uterine artery was not identified. All patients received 600-μm +/− 75-μm HydroPearl Microsphere embolization particles as part of the UAE procedure, with additional vials of larger particles administered as needed to achieve the desired endpoint (Table 1). Eight of the 18 patients (44.4%) required HydroPearl particle upsizing to 800-μm or larger to achieve the desired endpoint of 3–5 cardiac cycles of stasis within the uterine artery.

**Table 1:** Characteristics and clinical outcomes of patients who underwent UAE for the treatment of symptomatic uterine fibroids with HydroPearl Microspheres. Max size of dominant fibroid was the maximum dimension in centimeters reported for the largest fibroid on the pre-procedural MRI report. Bulk symptoms include pelvic fullness, pelvic heaviness, pelvic pain, constipation, dyspareunia, and urinary symptoms

Patient Number	Age	Race	Parity	BMI	Number of Fibroids	Max size of dominant fibroid (cm)	Total number of vials / size of microspheres (μm)	Symptoms	Menorrhagia Symptoms Improved?	Bulk Symptoms Improved?	Last clinical follow-up (days post-procedure)	Complications
1	54	White	3	26.6	1	8.4	2/600	Menorrhagia, Bulk	Yes	Yes	114	Amenorrhea
2	54	Black	0	27.5	> 6	8.4	4/600	Menorrhagia	Yes	–	188	
3	49	White	Unknown	24.2	2	9	3/600, 1/800	Menorrhagia, Bulk	Yes	Yes	104	Amenorrhea
4	31	Black	1	42	4	5.5	4/600, 0.5/800	Menorrhagia, Bulk	Yes	Yes	317	
5	47	Black	2	30.7	1	3.6	1/600	Menorrhagia	No	–	78	
6	44	Black	3	43.4	4	3.1	2/600	Menorrhagia, Bulk	No	No	99	
7	38	White	0	31.7	1	8.8	3/600	Menorrhagia, Bulk	Yes	Yes	92	
8	39	Black	4	28.3	> 6	3	3/600, 2/800	Menorrhagia	Yes	–	310	
9	43	Black	1	22.4	> 6	10.1	4/600, 2/800	Menorrhagia, Bulk	Yes	Yes	204	
10	47	White	3	35	1	4	Unknown/600	Menorrhagia	Yes	–	98	Amenorrhea
11	53	Black	1	28.3	> 6	6.3	3/600	Menorrhagia	Yes	–	92	Amenorrhea
12	54	Black	0	40.2	> 6	9.8	3/600, 6/800, 1/1100	Bulk	–	Yes	232	
13	47	Black	0	24.3	> 6	6.6	4/600	Menorrhagia, Bulk	Yes	Yes	105	
14	41	Black	0	55.3	1	8.5	4/600	Menorrhagia, Bulk	No	Yes	79	
15	42	Black	1	43.3	1	10.9	4/600, 3/800	Bulk	–	Yes	183	
16	43	Black	2	65.5	1	8.3	4/600	Menorrhagia, Bulk	Yes	Yes	93	
17	42	Black	0	45.3	> 6	9.6	8/600, 7/800, 1/1100	Menorrhagia, Bulk	Yes	Yes	113	
18	47	American Indian	2	22	2	11.8	5/600, 5/800, 2/1100	Menorrhagia, Bulk	Yes	Yes	100	
**Mean (SD)**	**45.3 (6.2)**		**1.4 (1.3)**	**35.3 (12.0)**		**7.5 (2.7)**					**144.5 (76.7)**	

*BMI* body mass index, *SD* standard deviation

**Fig. 1:**
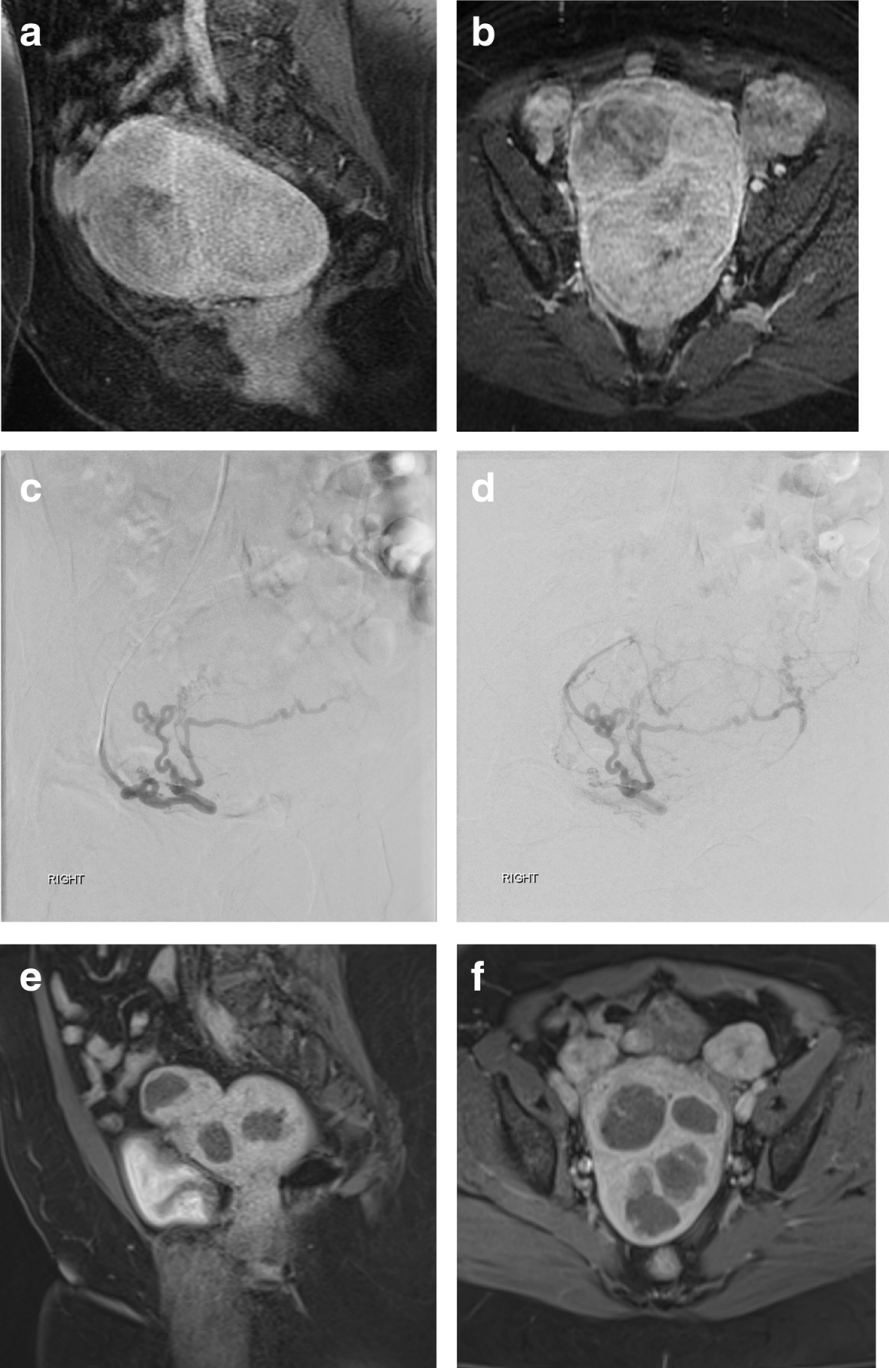
**a**-**f** Patient 12 is a 54-year old woman with bulk symptoms including pelvic pressure and heaviness, urinary frequency and urgency, and constipation. Sagittal (**a**) and axial (**b**) T1 post-contrast MRI images demonstrate a number of large uterine fibroids with internal enhancement. Digital subtraction angiography (**c**, **d**) as part of the UAE procedure demonstrates an enlarged, tortuous right uterine artery, with opacification of multiple masses. The left uterine artery was not identified. Sagittal (**e**) and axial (**f**) T1 post-contrast MRI images obtained 232 days after UAE demonstrates near complete necrosis of the majority of the uterine fibroids with decreased size and lack of internal enhancement. The patient had resolution of symptoms

Of the 18 patients who underwent UAE, 16 initially presented with symptoms of menorrhagia (88.9%). Thirteen of the 18 patients complained of bulk-type symptoms related to their uterine fibroids at initial presentation (72.2%). On clinical follow-up at least 3 months post-procedure, 13 of 16 patients (81.3%) reported improvement of bleeding symptoms. Of the 13 patients who presented with bulk-type symptoms, 12 (92.3%) reported symptomatic improvement on follow-up (Table 1). Overall, the most recent clinical follow-up documentation by the interventional radiologist who performed the procedure occurred at an average of 144.5 days (SD 76.7), or 4.8 months, post-procedure (range, 78 to 317 days). All patients had cross-sectional MRI imaging prior to undergoing uterine artery embolization. The interventional radiologist did not routinely obtain post-procedure pelvic MRIs unless there was an incomplete clinical response. Patient 12 underwent MRI with and without contrast at 232 days post-procedure in follow-up of a renal angiomyolipoma, which was incidentally discovered on MRI prior to UAE and was subsequently embolized in a separate session. Post-procedure contrast enhanced T1 weighted pelvic MRI images demonstrate near-complete necrosis of the uterine fibroids with lack of internal enhancement noted for the majority of the fibroids (Fig. 1).

Review of the clinical follow-up documentation did not reveal any major complications attributable to the UAE procedure. However, the UAE procedure precipitated amenorrhea in 4 of the 18 patients (22.2%, SIR class A). These 4 patients had an average age of 51 years (SD 3.3 years) at the time of the procedure (range, 47–54 years).

## Discussion

This small retrospective study summarizing our initial experience using HydroPearl Microspheres (Terumo Interventional Systems) as the embolic agent for UAE for the treatment of symptomatic uterine fibroids found that 81.3% of patients with menorrhagia experienced improvement in symptoms at follow-up an average of 136.6 days post-procedure (SD 77.4 days), or 4.6 months. For patients initially presenting with bulk-type symptoms, 92.3% experienced symptom improvement at an average of 141.1 days post-procedure (SD 71.4 days), or 4.7 months. The observed rate of clinical response that we observed, for both menorrhagia and bulk symptoms, for HydroPearl Microsphere particles was overall similar to other previously studied embolic agents for UAE. These include polyvinyl alcohol with or without gelfoam, polyphosphazene- coated hydrogel microspheres (Embozene, Varian Medical, Palo Alto, CA), tris-acryl gelatin spheres (Embospheres and Embogold, Merit Medical, South Jordan, UT), and gelatin sponge particles/porous gelatin particles (Toda et al., 2016; Salehi et al., 2015; Stampfl et al., 2011; Joffre et al., 2004; Lohle et al., 2006).

No major complications attributable to the UAE procedure were identified in this small series. The only complication documented in the EMR was the precipitation of amenorrhea in 4 of the 18 treated patients (22.2%). This is a known complication of UAE, particularly for older patients (Hamoda et al., 2015; Wozniakowska et al., 2013; Goodwin et al., 2008). These patients had an average age of 51 years, which is the average age of the onset of menopause for women in the United States (Bain et al., 2018).

HydroPearl Microsphere particles (Terumo Interventional Systems) have several unique characteristics that could prove beneficial for the treatment of symptomatic uterine fibroids with UAE. The particles are tightly calibrated, with at least 90% of the particles falling within the size range described. Compared to other spherical embolic particles such as Embosphere and Embozene, the tightly calibrated size distribution of HydroPearl Microspheres have demonstrated numerous benefits in in-vivo animal experiments. First, embolization with the HydroPearl Microspheres resulted in tighter luminal packing, ultimately causing greater tissue necrosis (Dasnurkar, 2015). The precisely calibrated size of these particles could also decrease non-target embolization, resulting in decreased damage to healthy tissues (Dasnurkar, 2015). HydroPearl Microspheres were found to be compressible, maintaining their spherical shape even after being pushed through a microcatheter, allowing for predictable embolization (Dasnurkar, 2015). This compressibility also allows for larger particles, such as 1100-μm particles, to be successfully delivered through a 2.8-Fr microcatheter if needed. We utilized the technique of administering 1100-μm HydroPearl particles through a 2.8 french Progreat microcatheter (Terumo Interventional Systems) in this small series, which was successful, but required frequent flushing of the microcatheter. Each size of the HydroPearl Microspheres has a unique particle color to ensure that the interventionalist is using the desired size. The color of the particles also allows for easy visualization to ensure adequate suspension of the particles prior to embolization, which could help to achieve an ideal embolization endpoint.

This retrospective study has limitations. The sample size of the study was small, and thus a determination of the safety and efficacy of this embolic agent for UAE for the treatment of symptomatic uterine fibroids cannot be made. The data reported in this study were obtained from EMR documentation of clinical follow-up either by telephone or in clinic. Standardized questions were not posed to each patient during these interactions, which could have introduced bias into this small study. Symptom questionnaires were not utilized in these clinical visits. Another major weakness of this study is that there was no control group, which would have allowed for direct comparison to other embolic agents. While all patients were initially scheduled for 1- and 3-month in-person clinic follow-up visits, many patients canceled or rescheduled their appointments, resulting in follow-up at variable times post-procedure and a few follow-up visits being conducted over the telephone. Some, but not all, patients had a documented 6-month or later follow-up, at the physician’s discretion. An additional limitation is that there were no specific inclusion criteria, as all patients that underwent embolization for fibroids using HydroPearl Microspheres within a certain time period were included. Consequently, patients had variable morphologies of their uterine fibroids (both size and location) that may have impacted the results.

## Conclusions

Our initial experience with using the HydroPearl Microsphere embolization particle was positive. There are several attributes of the embolic that make it appealing for UAE, such as tight size calibration and compressibility, allowing for easy delivery of large particles via a microcatheter. We found that in this small retrospective study, symptomatic improvement for menorrhagia and bulk-type symptoms at least 3 months post-procedure was comparable to other embolic agents as reported in the literature. Randomized clinical studies are needed in order to directly compare the clinical outcomes of the HydroPearl Microsphere to other embolic agents for UAE for uterine fibroids.

## Data Availability

All data generated or analysed during this study are included in this published article.

## Abbreviation

UAE: Uterine artery embolization
